# Supplementary material for: A Metal-Free, Disulfide Oxidized Form of Superoxide Dismutase 1 as a Primary Misfolded Species with Prion-Like Properties in the Extracellular Environments Surrounding Motor Neuron-Like Cells
Source: Int J Mol Sci. 2021 Apr 16;22(8):4155. doi: 10.3390/ijms22084155 (PMC8074096; doi:10.3390/ijms22084155)
Supplement: Supplementary file 1 [file ijms-22-04155-s001.zip › ijms-1174137-supplementary.pdf]

Supplementary Figure S1

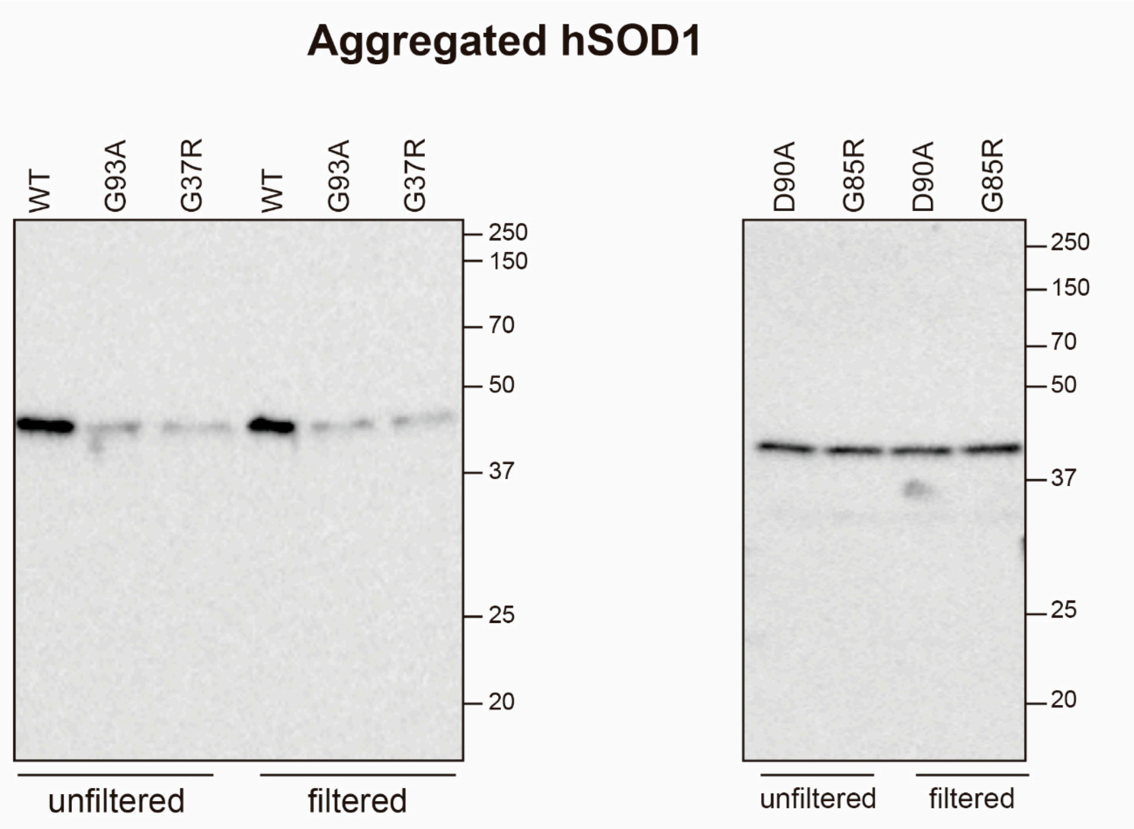

Supplementary Figure S1: Aggregated hSOD1 does not exist in the conditioned medium from our motor neuron models of ALS. The conditioned medium from NSC-34 cells transfected with hSOD1^{WT}-GFP or the ALS-linked hSOD1-GFP was treated with 100 mM iodoacetamide to block artificial oxidation of the thiol group of proteins. The concentrated conditioned medium was filtered with a 0.22 μm membrane filter to remove large hSOD1 aggregates and analyzed using Western blotting with anti-SOD1 antibody. Note that no aggregated hSOD1 was observed in the medium from our motor neuron models of ALS.
